# Cytologic and Histologic Findings of Extrapleural Solitary Fibrous Tumor: Report of Two Cases

**DOI:** 10.1002/dc.70045

**Published:** 2025-11-21

**Authors:** Michael Tyler, Katsiaryna Khatskevich, Chadi Hajar, Jack Yang, Hao Liu

**Affiliations:** ^1^ Department of Pathology and Laboratory Medicine Medical University of South Carolina Charleston South Carolina USA

**Keywords:** cytology, fine needle aspiration (FNA), pancreas, parotid gland, signal transducer and activator of transcription 6 (STAT6), solitary fibrous tumor (SFT)

## Abstract

Solitary fibrous tumors (SFT) are a rare neoplasm of mesenchymal origin. SFT was previously described primarily in the pleura and meninges; however, extrapleural and extra‐meningeal SFT have been reported in almost every anatomic site and account for up to 40% of cases. The most significant histologic findings of SFT include spindle cell proliferation in a “pattern‐less pattern”, dilated and branching “staghorn”‐like vasculature, and ropey collagen deposition. However, these findings are not consistently present in every case of SFT and may also be seen in other diseases. SFT has a characteristic NAB2::STAT6 gene fusion and nuclear overexpression of STAT6. The rarity of the disease, broad range of differential diagnoses, and wide spectrum of cytomorphological and histologic findings make the diagnosis of extrapleural SFT, especially on a fine needle aspiration (FNA) specimen, challenging. Recognizing and including this entity in the differential is necessary before the final diagnosis may be achieved through proper immunohistochemical and molecular workup. In this paper, we present two cases of extrapleural SFT with unusual locations: the first is a primary SFT present in a parotid gland and the second is a metastatic SFT present as two solid pancreatic masses.

## 1 Introduction

Solitary fibrous tumors (SFTs) are a rare neoplasm of mesenchymal origin and are presumed to arise from adult mesenchymal stem cells [1]. With the identification of the NAB2::STAT6 gene fusion, and recognition of STAT6 as a sensitive and specific marker for SFT, the diagnosis of this neoplasm has become a more straightforward process [2]. Despite these advancements, however, the histologic differential diagnoses for these tumors remain broad (Table 1) and may vary on a case‐by‐case basis with the given location of the tumor.

**TABLE 1:** Common differential diagnosis for a spindle cell lesion.

Diagnosis	Cytologic findings	Histologic findings	Positive immunohistochemical stains	Molecular findings
SFT	Plump spindle cells with bland nuclei, either dispersed or embedded in metachromatically staining ropy collagen	Plump spindle cells Ropy collagen Branching or staghorn‐shaped vessels; Perivascular hyalinization common	STAT6, CD34, BCL‐2, CD99	*NAB2::STAT6*
Schwannoma	Tightly cohesive groups of well‐organized spindle cells arranged in fascicles; few, if any, single cells Some nuclei may be wavy, with pointed or “fishhook” ends May have random nuclear pleomorphism (Ancient Change), but no mitotic activity	Biphasic with hypercellular (Antoni A) and hypocellular (Antoni B) areas Verocay bodies Hyalinized blood vessels	S100 and SOX10 Diffuse and strong positive. CD34 (variable)	Mutations in *NF2*
MPNST	Densely cellular fascicles of tapered spindle cells with wavy or comma shaped nuclei Abundant mitoses, and widely variable degrees of pleomorphism	Hypercellular spindle cells in herringbone or storiform patterns Tapered nuclei are hyperchromatic and crowded with abundant mitoses	S100 and SOX10 usually patchy positive (except for epithelioid MPNST)	*50% associated with NF1*
Leiomyoma	Cohesive, bland spindle cells with blunted nuclei in fascicles Few, if any, mitoses; smooth nuclear contours Moderate amount of eosinophilic cytoplasm	Spindle cells with brightly eosinophilic cytoplasm and blunt‐ended nuclei (“cigar nuclei”) Perpendicularly oriented fascicles Perinuclear vacuoles	SMA, desmin, caldesmon, ER, PR, AR (AR ~30%)	*Somatic mutations in the MED12 gene* *HMGA2*
Leiomyosarcoma	Round to spindle cells with blunted nuclei Increased mitotic activity, pleomorphism, and atypia as compared to leiomyoma	Spindle cells with blunt nuclear ends (“cigar nuclei”) Moderate to severe pleomorphism and high mitotic activity	SMA, desmin, caldesmon Diffuse p53(+)	Mutations in *TP53, RB1*, and *PTEN*. Loss of *CDKN2C*
Synovial sarcoma	Monomorphic variant composed of bland, uniformly dispersed spindle cells with scant cytoplasm Biphasic variant also includes epithelial cells, demonstrates a distinctive pattern of alternating areas of cellular dispersion and clustering	Monophasic variant features dense, evenly cellular sheets or vague fascicles of bland monomorphic spindle cells with scanty cytoplasm Biphasic variant additionally features epithelial structures that form glands, ducts, or nests	TLE1, pancytokeratins, EMA, BCL‐2 SS18‐SSX chimeric protein (E9X9V) and C‐terminus of SSX (E5A2C)	*SS18::SSX*
Desmoid fibromatosis	Uniform spindle cells arranged in slender fascicles with low cellularity and occasional crush artifact Rare mitotic activity Fragments of dense collagenous stroma	Uniform, spindled to stellate cells in long, “sweeping” fascicles or loose, vague storiform arrays Collagenous stroma often demonstrates subtle rims of perivascular clearing Can have variable mitotic activity, but lack nuclear pleomorphism	Nuclear beta catenin(+) in 75% cases SMA, MSA	*CTNNB1* and *APC*
DFSP	Dense clusters of uniform spindle cells with collagenous or myxoid stroma Arranged in a storiform or whorled pattern Entrapment of adipose tissue with fascicles of spindle cells is a helpful finding.	Storiform growth of uniform spindled tumor cells Characteristic honeycombing infiltration of subcutaneous fat or grow along fibrous septa	CD34: Diffuse positive	*COL1A1::PDGFB*
GIST	Fairly cohesive and well‐organized fascicles of uniform spindled or epithelioid cells, with occasional single cells Spindle cells feature scant inconspicuous fibrillary cytoplasm Epithelioid cells feature moderate to abundant eosinophilic cytoplasm	Spindled, epithelioid, or mixed‐type morphology Majority feature a predominantly spindled morphology, featuring uniform fusiform cells arranged in sheets or fascicles, May also show diffuse myxoid change or prominent stromal hyalinization/sclerosis	DOG1, CD117/c‐KIT CD34 is frequently positive	*KIT* or *PDGFRA* mutations

Abbreviations: DFSP, dermatofibrosarcoma protuberans; ER, estrogen receptor; GIST, gastrointestinal stromal tumor; MPNST, malignant peripheral nerve sheath tumor; MSA, muscle specific actin; PR, progesterone receptor; SFT, solitary fibrous tumor; SMA, smooth muscle actin.

While originally described as a pleural‐based tumor, SFT has since been recognized to also arise in many additional sites including the lungs, peritoneum, gastrointestinal tract, mediastinum, and head and neck regions [3, 4, 5, 6, 7, 8, 9]. Within the head and neck, the sinonasal tract, orbit, and oral cavity are among the most commonly reported sites [10]. In recent years, there has also been growing recognition of this tumor occurring in the parotid gland and pancreas [11, 12, 13, 14, 15, 16, 17, 18].

In the literature, epidemiologic data on SFT is often divided into meningeal and extrameningeal (or “soft tissue”) cases. Meningeal‐based cases occur with an incidence of 0.38 per 1,000,000 people while extrameningeal cases occur with an overall incidence of 0.61 per 1,000,000 people [19, 20]. Cases are known to occur throughout adulthood, with the median age at diagnosis being 50–60 years [21]. While cases are fairly evenly distributed among gender, patients of Asian/Pacific Islander heritage have been noted to have a slightly higher incidence rate compared to other ethnic groups; but, interestingly, this has only been observed among meningeal cases [19, 20].

In this article, we present two cases of SFT with unusual clinical presentations with a focus on important cytologic and histologic features that are often key to establishing the diagnosis.

## 2 Case Report

### 2.1 Case #1

A 50‐year‐old female smoker with no malignant history presented with a right parotid mass for approximately 18 months. The mass would subtly wax and wane in size and would cause intermittent facial tenderness. CT imaging showed the mass measuring 6.1 cm in greatest dimension.

**FIGURE 1:**
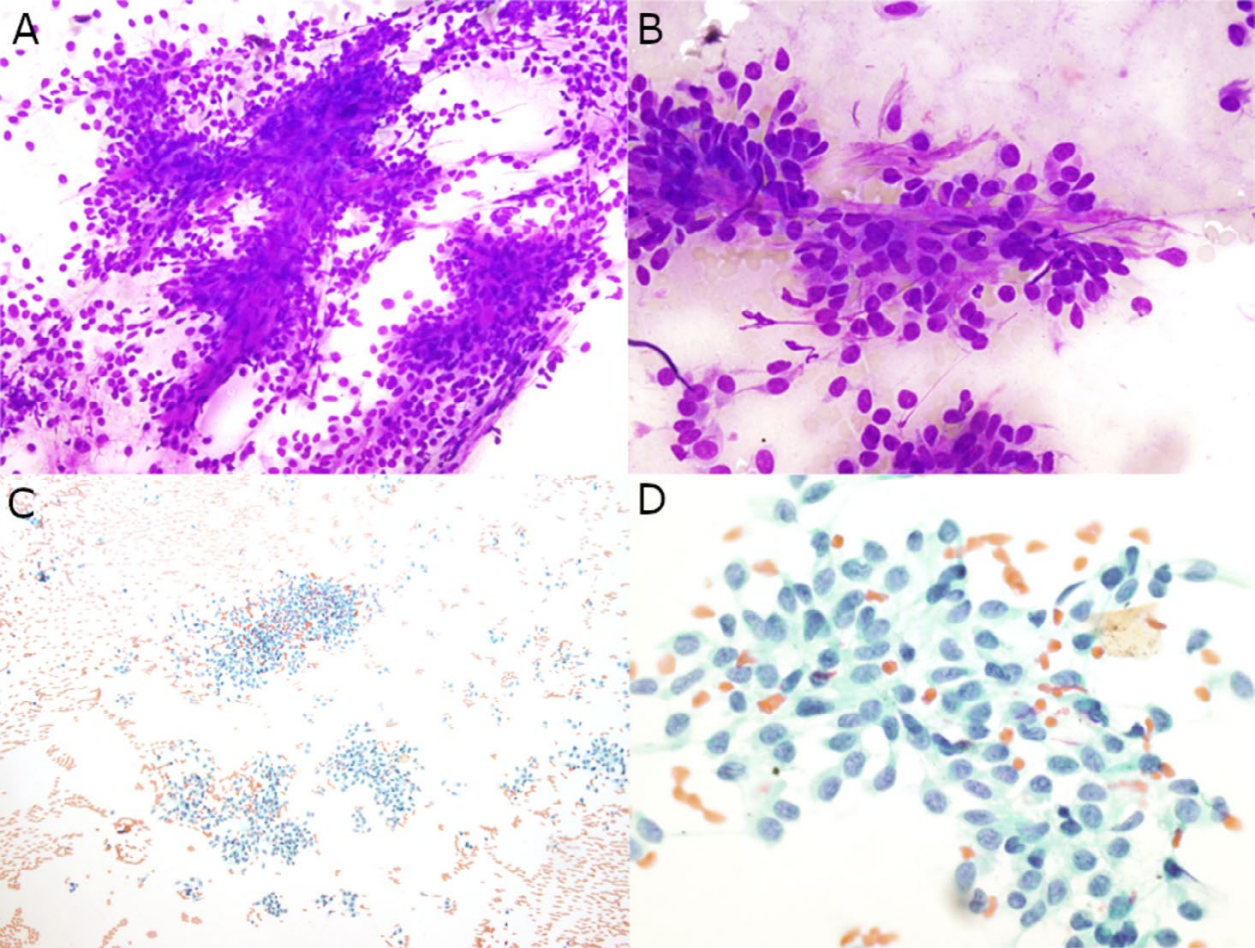
Cytologic findings of the parotid mass fine needle aspirate. The aspirate smear of the parotid mass comprises of tumor cells with oval to short spindle shaped nuclei, mild nuclear pleomorphism, fine granular chromatin, and small amounts of cytoplasm present singly and in clusters associated with scant collagenous material in a background of naked nuclei and blood. (A and B) Diff‐Quik stained smears, original magnification ×200 and ×400, respectively. (C and D) Papanicolaou‐stained smears, original magnification ×100 and ×600, respectively.

An ultrasound‐guided fine needle aspiration (FNA) of the parotid mass was performed. The aspirate smear revealed a moderately cellular specimen comprising monotonous tumor cells with oval to short spindle‐shaped nuclei, smooth nuclear contours, mild nuclear pleomorphism, fine granular chromatin, inconspicuous pinpoint nucleoli, and small amounts of cytoplasm with indistinct cell borders. The tumor cells were present singly, in clusters, and in fascicles associated with scant, ropy collagenous material in a background of naked nuclei and blood (Figure 1). These findings were reported as consistent with a salivary gland neoplasm. Based on the location of the tumor in a salivary gland and cytology, the differential diagnoses included pleomorphic adenoma, myoepithelioma, and other salivary gland tumors. A parotidectomy was subsequently performed.

**FIGURE 2:**
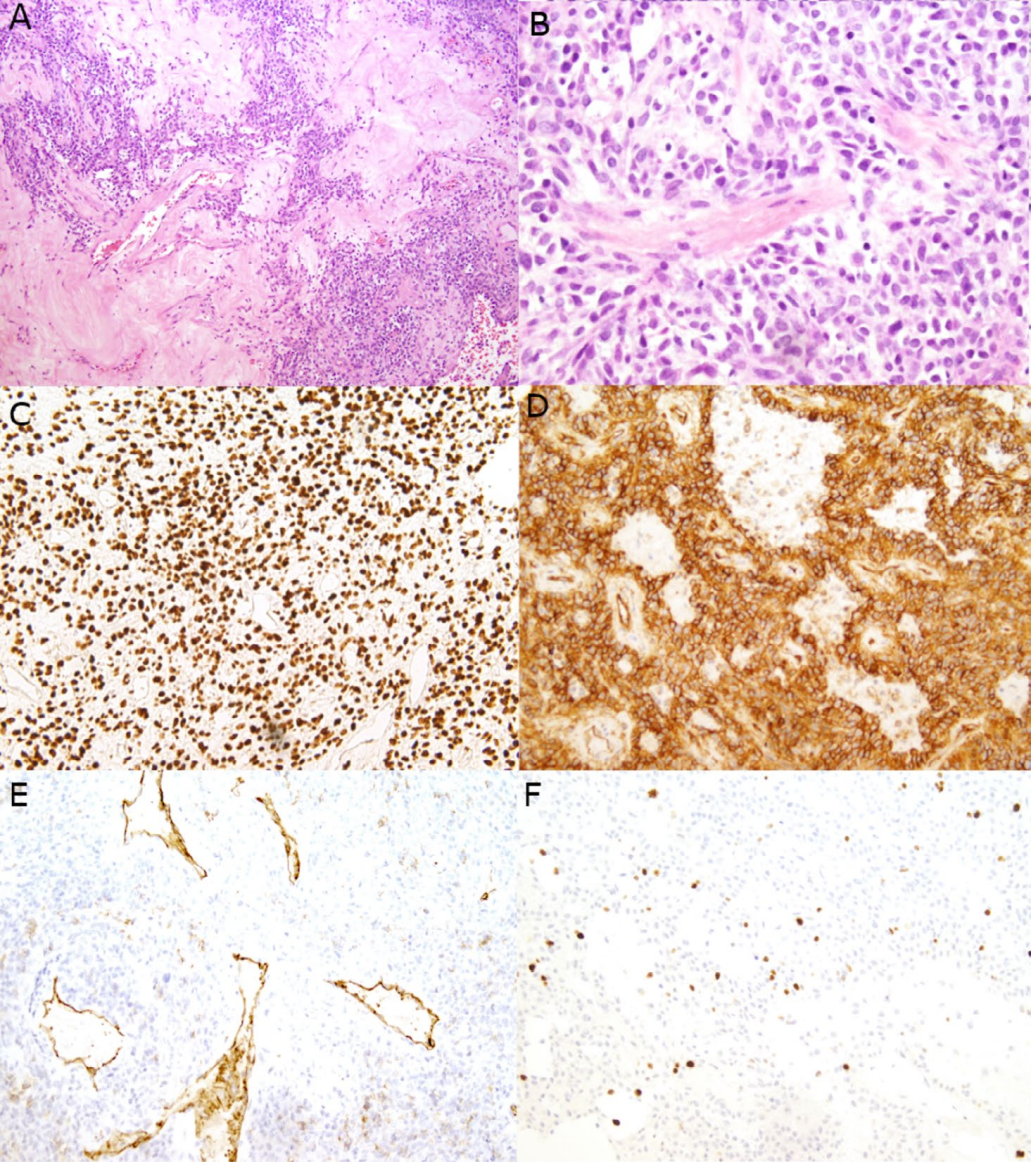
Histology and immunohistochemical profile of the parotid mass core biopsy specimen. Core biopsy shows alternative hypercellular and hypocellular areas and branching staghorn vessels with hyalinized vessel walls. Oval to short spindle tumor cells with mild irregular nuclear contours are present with collagen material. (A and B) H&E, original magnification ×100 and ×400, respectively. Immunohistochemical staining shows tumor cells are positive for STAT6 (C, ×200) and CD34 (D, ×200). CD31 (E, ×200) highlights the branching vessels and are negative for tumor cells. Ki67 highlights approximately 4% of tumor cells (F, ×200).

On histology, the parotid gland mass showed alternating hypercellular and hypocellular areas as well as branching “staghorn” vessels with thickened, hyalinized vessel walls. Oval to short, spindled tumor cells with mild pleomorphism were present with associated collagenous material in a “pattern‐less” pattern (Figure 2). No significant nuclear atypia or necrosis was noted and only minimal mitotic activity was identified. The differential diagnosis of this spindle cell neoplasm of the parotid gland was broad and a series of immunohistochemical (IHC) stains were performed. Tumor cells were negative for AE1/AE3, CD117, S100, and SOX10. CD31 and SMA highlighted the branching vessels and were negative in the tumor cells. Tumor cells were positive for CD34, CD99, BCL2, and showed strong and diffuse nuclear positivity for STAT6. Ki67 highlighted approximately 4% of tumor cells. The final diagnosis of SFT was made. The patient underwent adjuvant radiotherapy due to positive surgical margins and showed no evidence of recurrence in the first few months following her surgery.

### 2.2 Case #2

A 53‐year‐old male smoker presented to the hospital with complaints of nausea, vomiting, dizziness, and abdominal pain localized to the lower abdominal quadrants. As part of his work‐up, he underwent a CT abdomen/pelvis and subsequent MRI which were remarkable for two large pancreatic masses; one mass measured up to 7.9 cm and was located near the junction of the pancreatic head and body while the other mass measured up to 6.7 cm and was located in the pancreatic tail. A subsequent follow‐up PET CT showed mild hypermetabolic activity in both pancreatic masses as well as a 1.3 cm lung nodule.

His past medical history was significant for a dural‐based left occipital mass reportedly diagnosed as a hemangiopericytoma, which had been previously treated by craniotomy and postoperative radiation twice: once 15 years prior and again the previous year. The pathology report on the most recent resection was consistent with a recurrent SFT, WHO Grade 3. Unfortunately, this past medical history was not available initially. Gastroenterology was consulted for endoscopic ultrasound‐guided FNA biopsy of both pancreatic masses to establish a diagnosis.

**FIGURE 3:**
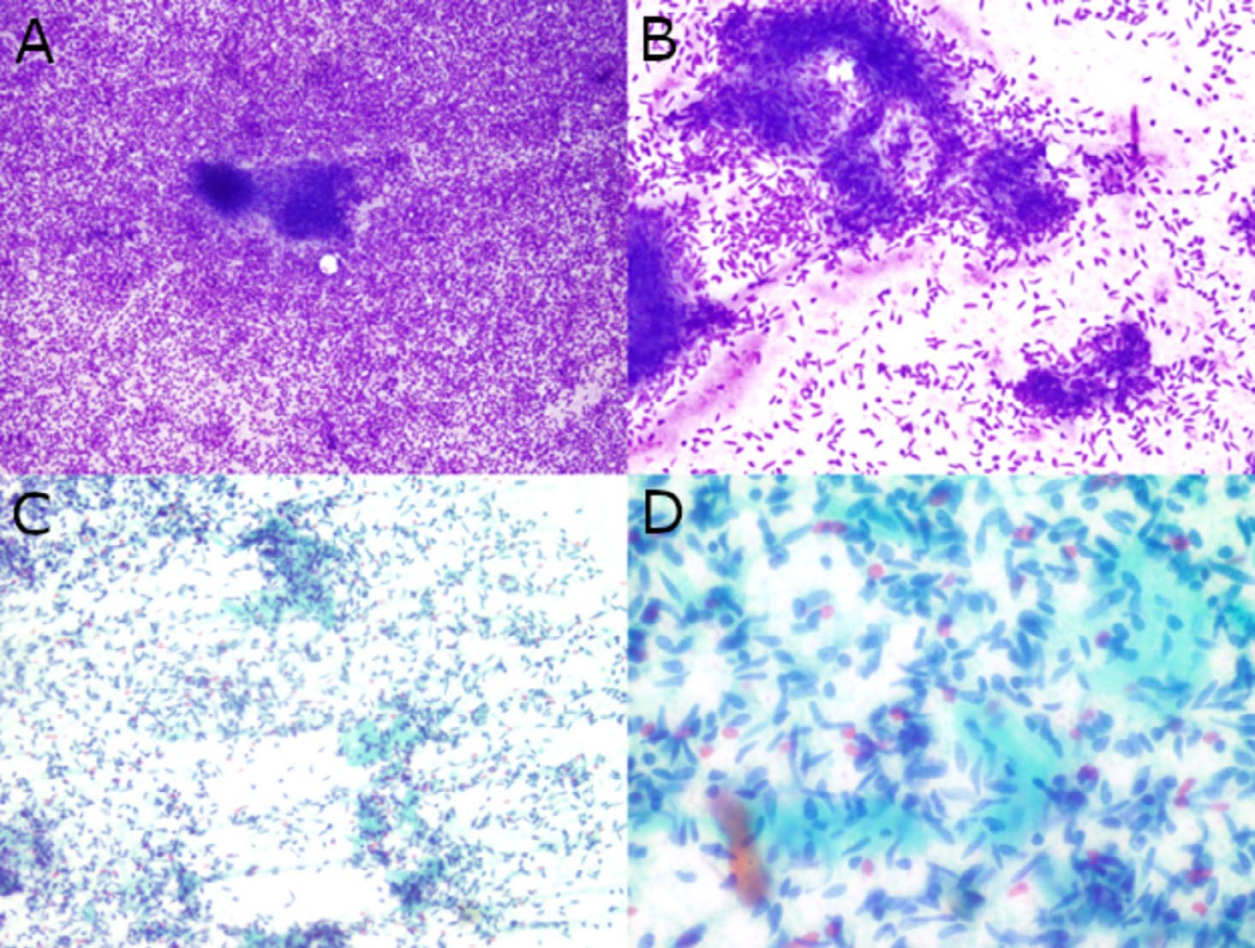
Cytologic findings of the pancreatic head mass fine needle aspirate. The aspirate smear of the pancreatic head mass was a hypercellular specimen comprising of monomorphic spindle shaped tumor cells with fine chromatin, mild nuclear pleomorphism, and scant cytoplasm present singly and in loose clusters, associated with scant collagenous material in a background of naked nuclei and blood. (A and B) Diff‐Quik stained smears, original magnification ×40 and ×200, respectively. (B and D) Papanicolaou‐stained smears, original magnification ×200 and ×600, respectively.

**FIGURE 4:**
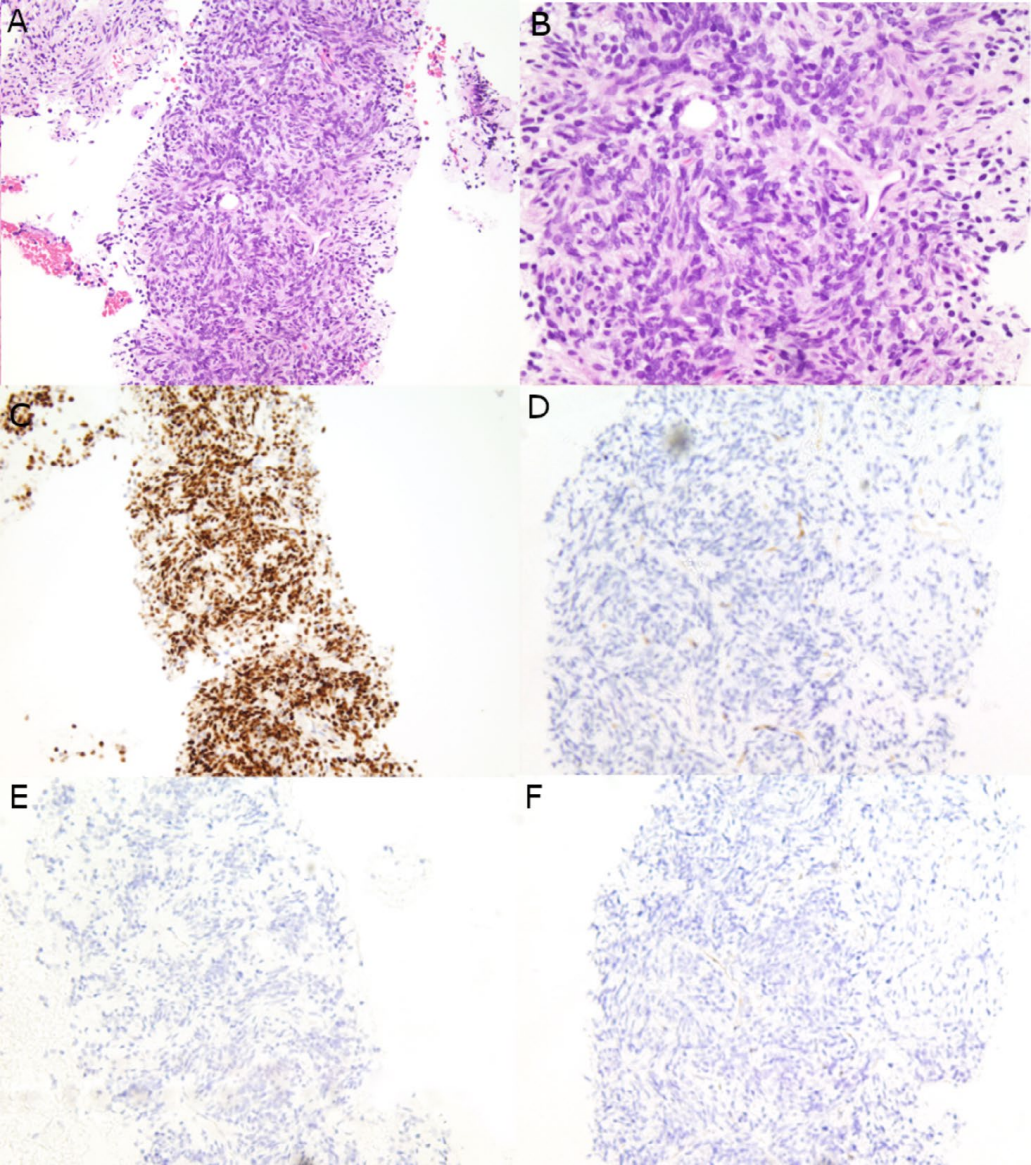
Histology and immunohistochemical profile of the pancreatic head mass core biopsy specimen. Core biopsy of the pancreatic head mass shows spindle shaped neoplastic cells organized in alternative hypercellular and hypocellular areas with branching staghorn vessels. (A and B) H&E, original magnification: ×200 and ×400, respectively. Immunohistochemical staining reveals tumor cells to be positive for STAT6 (C, ×200), but negative for CD117 (D, ×200), INSM (E, ×200), and DOG1 (F, ×200).

FNA of both masses yielded hypercellular specimens with similar cytologic findings: monotonous neoplastic cells with oval to short, spindle‐shaped nuclei, mild nuclear pleomorphism, fine chromatin, occasional inconspicuous nucleoli, and small amounts of cytoplasm. The tumor cells were present both singly and in a meshwork of fascicles with associated collagenous material in a background of naked nuclei and blood (Figure 3). These findings were ruled as consistent with a spindle cell neoplasm. Tissue biopsies of both masses demonstrated spindle‐shaped, mildly pleomorphic neoplastic cells in alternating hypercellular and hypocellular areas with branching, ectatic “staghorn” vessels and hyalinized collagen (Figure 4). The bland and relatively uniform neoplastic cells were arranged in a “pattern‐less” pattern. No necrosis or increased mitoses were identified. Without knowing the patient's previous history of SFT, a broad differential including gastrointestinal stromal tumor (GIST), schwannoma, smooth muscle neoplasm, synovial sarcoma, and neuroendocrine tumor (NET) was considered in addition to SFT. IHC showed that the neoplastic cells were positive for STAT6 and negative for CD117, DOG1, synaptophysin, and INSM1 in both specimens. A careful search of the patient's medical history later revealed his past medical history of recurrent SFT. With this additional information, a diagnosis of metastatic SFT was favored.

Following diagnosis, follow‐up brain and whole‐spine MRIs were performed to assess for possible foci of metastatic recurrence. Numerous small enhancing nodules were found at the prior craniotomy site, likely representing recurrent disease, and a 2.5 cm enhancing dural‐based lesion was found along the olfactory groove with possible extension into the adjacent ethmoid air cells, possibly representing a new focus of the patient's primary SFT.

## 3 Discussion

The cytology findings of SFT in our cases included variably cellular specimens with tumor cells present singly and in loose clusters in a scant collagenous or myxoid background. The monomorphic tumor cells exhibited spindled to oval nuclei, mild nuclear pleomorphism, fine chromatin, inconspicuous nucleoli, and wispy cytoplasm. Delicate networks of branching blood vessels were occasionally present [22, 23]. These cytology findings also correlated with the classic histology findings of SFT: a spindle cell proliferation arranged in a “pattern‐less pattern” in a background of variably collagenous stroma, thin branching “staghorn” vessels and focal perivascular fibrosis with varying degrees of myxoid change [24].

Due to the wide variety of anatomic sites in which SFT may arise, their relatively nonspecific spindled cytologic features, and the number of histologic patterns they may form, the differential diagnosis can be very broad. Depending on the site of the lesion, the differential diagnosis may include many spindle cell neoplasms, such as spindle cell lipoma, dedifferentiated liposarcoma, myofibroma, schwannoma, smooth muscle neoplasms, dermatofibrosarcoma protuberans (DFSP), and GIST (Table 1) [24]. Therefore, proper IHC staining plays an important role in the diagnosis of SFT. Previously, a combination of CD34, BCL‐2 and CD99 stains was used for diagnosing SFT, but these markers are neither specific nor sensitive. For example, up to 10% of the SFT cases are negative for CD34, especially in dedifferentiated SFT [24]. With the recognition of STAT6 as a sensitive and specific marker of SFT, the diagnosis of this neoplasm appeared to be a more straightforward process; yet recent publications suggest this could be a potential diagnostic pitfall as: (1) the STAT6 nuclear overexpression could be attenuated in SFT with high‐grade dysplastic features and (2) strong and diffuse nuclear positivity can be detected in more than 10% of dedifferentiated liposarcomas. It is also noteworthy that SFT can have focal or patchy positivity for keratin, PAX‐8 and SMA stains, which could be a diagnostic pitfall, especially in FNA specimens with a very limited number of neoplastic cells.

It can often be difficult to predict the malignant potential of SFT. There are multiple clinical and histologic features that have been shown to correlate with malignant potential such as patient age (> 55 years), tumor size (> 5 cm), increased cellularity, mitotic activity (≥ 2 mitoses/mm^2^), nuclear pleomorphism, necrosis (> 10%), and infiltration [21, 25, 26, 27]. However, many cases deemed to be low‐risk have been known to still recur, often returning with newly acquired high‐risk features. Recently, Tolstrup's group reported that the mitotic index, presence of necrosis, and Ki67 index are the most solid risk factors for recurrence. For most cases of SFT, the overall prognosis is generally very good. While anatomic considerations sometimes necessitate an individualized approach to treatment, most patients do very well with a treatment plan consisting of a combination of wide‐local excision and radiation therapy [28].

## Conflicts of Interest

The authors declare no conflicts of interest.

## Data Availability

Data sharing not applicable to this article as no datasets were generated or analyzed during the current study.
